# The Derivative of *Tripterygium wilfordii* Hook F—Kunxian Capsule, Attenuated Rheumatoid Arthritis: A Systematic Review and Meta-Analysis

**DOI:** 10.1155/2020/4178140

**Published:** 2020-08-12

**Authors:** Ya-Fei Liu, Zhe Zhang, Jun-Jun Zhang, Zhe Chen, Sheng-Hao Tu, Guo-Lan Xing

**Affiliations:** ^1^Department of Nephrology, The First Affiliated Hospital of Zhengzhou University, 1 Jianshe East Road, Zhengzhou, Henan 450052, China; ^2^Institute of Integrated Traditional Chinese and Western Medicine, Tongji Hospital, Tongji Medical College, Huazhong University of Science and Technology, 1095 Jiefang Avenue, Wuhan, Hubei 430030, China

## Abstract

The study aimed to explore the efficacy and safety of Kunxian Capsule (KXC) in the treatment of rheumatoid arthritis (RA). The randomized controlled trials (RCTs) comparing the effects of KXC in patients with RA were included in this study. Weighted mean differences (MDs) were calculated for net changes by employing Review Manager meta-analysis software. Nine RCTs were included in the systematic review with a total of 747 patients. The overall effects showed that KXC alone or combined with disease-modifying antirheumatic and drugs decreased tender joint counts (*P*=0.02, MD = −1.07, 95% CI: −1.95 to −0.18), shortened duration of morning stiffness (*P* < 0.0001, MD = −9.01, 95% CI: −13.08 to −4.93), lowered erythrocyte sedimentation rate (*P* < 0.00001, MD = −5.27, 95% CI: −6.78 to −3.77), and reduced C-reactive protein (*P* < 0.0001, MD = −5.04, 95% CI: −7.28 to −2.80). The most common adverse events were gastrointestinal disturbances and abnormal liver function. These results suggest that KXC is likely to be a more effective and safe candidate for treating RA compared with conventional therapies.

## 1. Introduction

Rheumatoid arthritis (RA) is an autoimmune disease inducing synovial inflammation, which is characterized by tenderness, swelling, morning stiffness, joint destruction, and deformity [1, 2]. The age-standardized prevalence and incidence rates are increasing, especially in countries such as Canada, Paraguay, and Guatemala. To reduce the ongoing burden of this condition, early diagnosis and treatment of RA are vital, especially among female patients [3].

The conventional therapies include nonsteroidal anti-inflammatory drugs (NSAIDs), glucocorticoids, and disease-modifying antirheumatic and drugs (DMARDs) [4, 5]. With the exploration of molecular mechanisms, biological agents have been wildly applied in the treatment of RA [6, 7]. However, owing to the adverse effects of conventional medications and high financial stress of biologicals [8], more effective and safe medications for RA are needed to be explored.

*Tripterygium wilfordii* Hook F (TWHF) has been applied in the treatment of RA in China for decades [9]. The previous research showed that TWHF could be as effective as synthetic DMARDs in the treatment of RA and the common adverse effects (AEs) were gastrointestinal discomfort, menstruation disorders, and amenorrhea [10]. Kunxian Capsule (KXC) is a derivative of TWHF and has little liver injury and reproductive toxics compared with TWHF [11]. Meanwhile, KXC has been employed in treating RA in clinical trials for a long time [12–14]. However, most of these clinical data origins from uncontrolled clinical trials or retrospective reports, and few multicenter clinical trials have been performed to verify the effects of KXC in the treatment of RA. Therefore, the scientific evidence confirming the efficacy and safety of KXC in treating RA remains unclear. Given these uncertainties, it is essential to perform a quantitative meta-analysis of the efficacy and safety of KXC in patients with RA. Consequently, we carried out the meta-analysis and systematic review.

## 2. Materials and Methods

The Preferred Reporting Items for Systemic Review and Meta-Analyses (PRISMA) statement [15] was applied to design and report the study.

### 2.1. Search Strategy

The following English databases were retrieved to confirm trials: PubMed, the Cochrane Library, and Clinical Trials.gov. In addition, the Chinese databases, such as the CNKI Database, Wanfang Database, VIP Database, CBM Database, and Chinese Clinical Trial Register were searched. All the databases were retrieved from their available dates of inception to the latest issue (June 2019).

Different search strategies were combined as follows. For the English databases, free text terms were employed, such as “kunxian capsule” and “rheumatoid arthritis” or “RA”. For the Chinese databases, free text terms were applied, such as “kunxian jiaonang” (which means Kunxian Capsule in Chinese) and “lei feng shi guan jie yan” (which means rheumatoid arthritis in Chinese). A filter for clinical trials was selected for English databases. To include an adequate number of trials, the reference lists of pertinent publications were also retrieved to identify additional studies.

### 2.2. Selection Criteria

Randomized controlled trials (RCTs) were included irrespective of blinding, publication status, or language. Studies were selected for reanalysis if they met the following criteria: (1) patients enrolled were diagnosed with RA, according to the 1987 or 2010 RA guidelines of the American Rheumatology Association; (2) in the intervention group, the participants took KXC alone or with other DMARDs; (3) in the control group, the participants took DMARDs; (4) duration was at least 12 weeks; and (5) outcomes were available.

Case reports, reviews, *in vitro* experiments, retrospective studies, or studies without control groups were excluded. For repeat studies, the authors were contacted to illuminate any ambiguities. The RCTs that lacked crucial data to allow for the calculation of net changes in outcomes and their variances from baseline to the endpoint were also excluded from the research. The literature was selected by two reviewers (YF Liu and Z Zhang) independently. The flowchart of the study selection has been generated in accordance with the PRISMA statement.

### 2.3. Data Extraction and Management

The data were extracted by two independent reviewers (Zhang and Chen), and contradictions were resolved by consensus or were judged by another reviewer.

The studies' quality was evaluated according to the Cochrane handbook 5.3. The risk of bias of the included trials was evaluated by the following items: (A) random sequence generation (selection bias); (B) allocation concealment (selection bias); (C) blinding of participants and personnel (performance bias); (D) blinding of outcome assessment (detection bias); (E) incomplete outcome data (attrition bias); (F) selective reporting (reporting bias); and (G) other bias.

The primary outcomes were tender joint count (TJC), swollen joint count (SJC), and duration of morning stiffness (DMS). The secondary outcomes included erythrocyte sedimentation rate (ESR), C-reactive protein (CRP), rheumatoid factor (RF), and anticyclic citrullinated peptide (CCP). Adverse events were also collected from the studies. For the trials that applied a three-armed group design, the outcomes of the groups were extracted if they satisfied the inclusion criteria. In terms of vagueness or absence in the publications of the outcomes, the authors were connected and related data has been extracted by consensus if the authors were unavailable.

### 2.4. Data Synthesis and Analysis

The effects of KXC on patients with RA were calculated as differences between the KXC group and the control group, utilizing the Review Manager meta-analysis software, version 5.3. To guarantee the credibility of the results, the net changes in all the outcomes were analyzed as the mean differences (KXC minus control) in changes (endpoint minus baseline) for parallel trials. The weighted mean differences and 95% confidence intervals (CIs) were calculated for continuous data. Heterogeneity was estimated through the chi-square test and Higgins *I*^2^ test. A fixed-effect model was applied when the studies were sufficiently alike (*P* > 0.10); otherwise, a random-effects model was applied. For the subgroup with high heterogeneity, a random-effects model was performed. A Z score was calculated to determine the overall effect, with a significance set at *P* < 0.05. Publication bias was analyzed by funnel plot if the number of the included studies ≥10.

Two subgroup analyses were performed to diminish the clinical heterogeneity: KXC compared with DMARDs and KXC plus DMARDs compared with DMARDs.

## 3. Results

### 3.1. Study Selection

**Figure 1:**
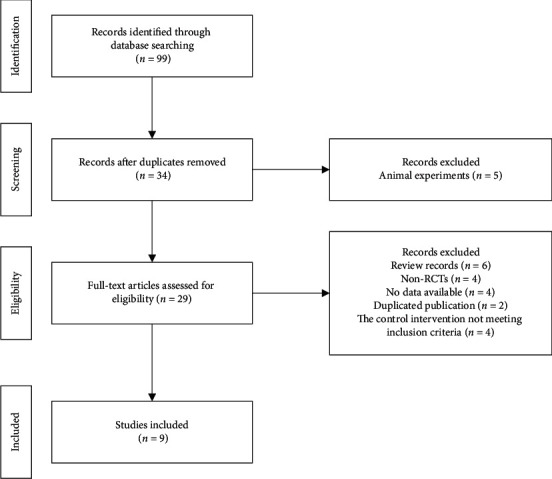
Flowchart of study selection.

The process of study selection was shown in Figure 1. After filtering, 5 animal experiments, 6 reviews, 4 studies without sufficient data, 2 studies with duplicated data, and 4 studies whose control intervention did not meet the inclusion criteria were excluded. According to the selection criteria defined in the Materials and Methods, 9 RCTs were included in the meta-analysis. The three-armed group design was applied in two of the studies. To prevent sample duplication, the data of the two groups were included [16, 17]. In one study, there were three groups: leflunomide alone, leflunomide plus KXC, and leflunomide plus methotrexate [16]. The data of leflunomide plus KXC versus leflunomide alone was extracted [16]. One study reported that KXC monotherapy was compared with methotrexate monotherapy or KXC plus methotrexate [17]. The data of KXC monotherapy versus methotrexate monotherapy was extracted [17]. In the KXC monotherapy subgroup, three studies were included [17–19]. In the combined therapy subgroup, six studies were included [16, 20–24]. The characteristics of the studies were indicated in Table 1. Together, 747 RA patients were included in the study.

### 3.2. Study Descriptions

The included studies were published as full texts between 2010 and 2019. All the RCTs were performed in China. All the studies were published in Chinese. Eight studies were conducted as single-center trials, and only one study was a multicenter trial [17].

### 3.3. Interventions and Controls

Three studies compared KXC with DMARDs (methotrexate or leflunomide) [17–19]. Six trials compared a cointervention of KXC and DMARDs (methotrexate, or leflunomide, or hydroxychloroquine) with DMARDs [16, 20–24]. NSAIDs were added to the control group in two studies [20, 21]. Different doses of KXC were administered in these studies. The KXC administration ranged from 0.9 g to 1.8 g per day. The total daily KXC was divided into two to three doses. The methotrexate administration ranged from 7.5 mg to 15 mg per week.

Except for one study with a duration of six months [19], all the other studies were 12 or 24 weeks. The outcomes were measured at 4 weeks and 12 weeks in two studies, and the outcomes of 12 weeks were extracted [17, 18]. The outcomes were determined at 4 weeks, 8 weeks, and 12 weeks in one study, and the results of 12 weeks were extracted [22]. The outcomes were detected at 4 weeks, 8 weeks, 12 weeks, and 24 weeks in one study, and the results of 24 weeks were extracted [16].

### 3.4. Quality of the Included Studies

**Figure 2:**
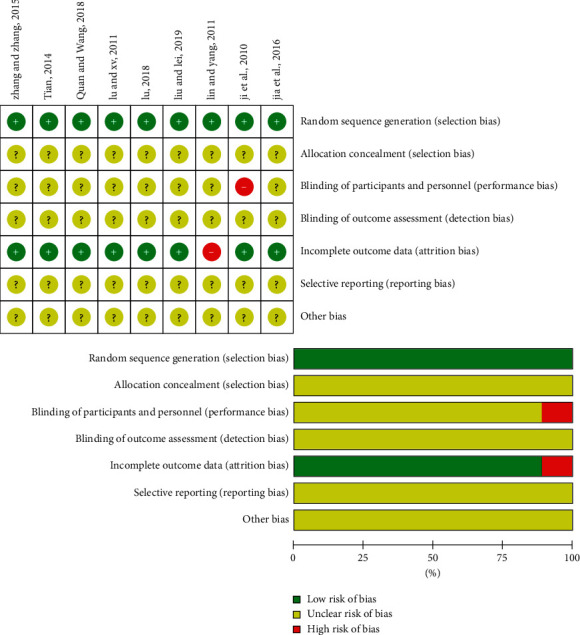
Risk of bias of the included studies.

As indicated in Figure 2, all the included trials were of low quality due to unclear randomization, deficient allocation concealment, inadequate blinding, and undescribed withdrawals and dropouts.

### 3.5. Publication Bias

As the number of included studies <10, the funnel plot was not conducted to estimate the publication bias.

### 3.6. Effects of Interventions

#### 3.6.1. The Outcome of TJC

**Figure 3:**
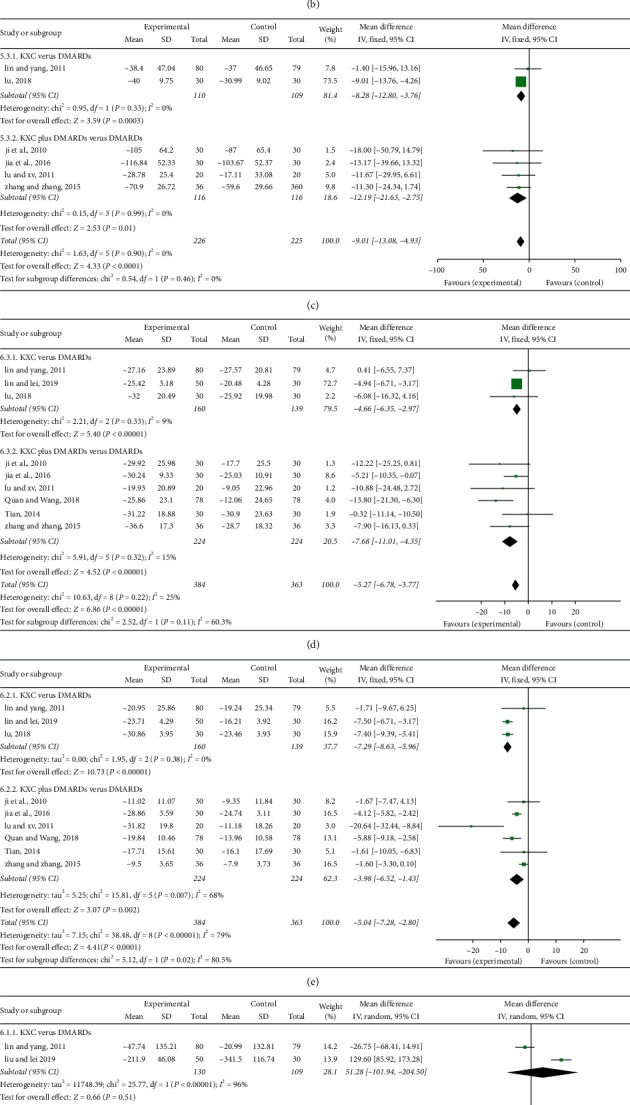
Forest plots for the outcomes. TJC: tender joint count; SJC: swollen joint count; DMS: duration of morning stiffness; ESR: erythrocyte sedimentation rate; CRP: C-reactive protein; RF: rheumatoid factor; and CCP: anticyclic citrullinated peptide.

As illustrated in Figure 3, only one study compared KXC with DMARDs in terms of TJC (involving 159 subjects), and there was no significant difference between the two groups (*P*=0.16, MD = −0.87, 95% CI: −2.08 to 0.34). Three studies compared KXC plus DMARDs with DMARDs regarding TJC (involving 160 subjects), and there was also no significant difference between the two groups (*P*=0.05, MD = −1.29, 95% CI: −2.60 to 0.02). However, the overall effect (involving 319 subjects) showed that KXC group was superior to the control group regarding lowering the TJC (*P*=0.02, MD = −1.07, 95% CI: −1.95 to −0.18).

#### 3.6.2. The Outcome of SJC

As shown in Figure 3, only one study compared KXC with DMARDs in terms of SJC (including 159 subjects), and there was no significant difference between the two groups (*P*=0.78, MD = −0.14, 95% CI: −1.11 to 0.83). Three studies compared KXC plus DMARDs with DMARDs regarding SJC (including 160 subjects) and there was a significant difference between the two groups (*P*=0.02, MD = −1.42, 95% CI: −2.57 to −0.27). Moreover, the overall effect (involving 319 subjects) demonstrated that there was no significant difference between the two groups (*P*=0.08, MD = −0.67, 95% CI: −1.42 to 0.07).

#### 3.6.3. The Outcome of DMS

As indicated in Figure 3, two studies compared KXC with DMARDs with regard to DMS (involving 219 subjects) and there was a significant difference between the two groups (*P*=0.0003, MD = −8.28, 95% CI: −12.80 to −3.76). Four studies compared KXC plus DMARDs with DMARDs regarding DMS (involving 232 subjects), and there was a significant difference between the two groups (*P*=0.01, MD = −12.19, 95% CI: −21.63 to −2.75). Moreover, the overall effect (involving 451 subjects) illustrated that KXC group was superior to the control group regarding reducing the DMS (*P* < 0.0001, MD = −9.01, 95% CI: −13.08 to −4.93).

#### 3.6.4. The Outcome of ESR

As illustrated in Figure 3, three studies compared KXC with DMARDs in terms of ESR (involving 299 subjects), and there was a significant difference between the two groups (*P* < 0.00001, MD = −4.66, 95% CI: −6.35 to −2.97). Six studies compared KXC plus DMARDs with DMARDs regarding ESR (involving 448 subjects), and there was a significant difference between the two groups (*P* < 0.00001, MD = −7.68, 95% CI: −11.01 to −4.35). Meanwhile, the overall effect (involving 747 subjects) showed that KXC group was superior to the control group regarding decreasing the ESR (*P* < 0.00001, MD = −5.27, 95% CI: −6.78 to −3.77).

#### 3.6.5. The Outcome of CRP

As shown in Figure 3, three studies compared KXC with DMARDs in terms of CRP (involving 299 subjects), and there was a significant difference between the two groups (*P* < 0.00001, MD = −7.29, 95% CI: −8.63 to −5.96). Six studies compared KXC plus DMARDs with DMARDs regarding CRP (involving 448 subjects), and there was a significant difference between the two groups (*P*=0.002, MD = −3.98, 95% CI: −6.52 to −1.43). Meanwhile, the overall effect (involving 747 subjects) showed that KXC group was superior to the control group regarding declining CRP (*P* < 0.0001, MD = −5.04, 95% CI: −7.28 to −2.80).

#### 3.6.6. The Outcome of RF

As illustrated in Figure 3, two studies compared KXC with DMARDs in terms of RF (including 239 subjects), and there was no significant difference between the two groups (*P*=0.51, MD = 51.28, 95% CI: −101.94 to 204.50). Five studies compared KXC plus DMARDs with DMARDs regarding RF (involving 408 subjects), and there was a significant difference between the two groups (*P*=0.0005, MD = −19.07, 95% CI: −29.76 to −8.39). However, the overall effect (involving 647 subjects) showed that there was no significant difference between the two groups (*P*=0.96, MD = −0.70, 95% CI: −31.79 to 30.40).

#### 3.6.7. The Outcome of CCP

As depicted in Figure 3, two studies compared KXC with DMARDs in terms of CCP (involving 239 subjects), and there was no significant difference between the two groups (*P*=0.27, MD = −49.16, 95% CI: −136.46 to 38.15). Two studies compared KXC plus DMARDs with DMARDs in terms of CCP (involving 216 subjects), and there was no significant difference between the two groups (*P*=0.24, MD = −74.53, 95% CI: −198.85 to 49.79). Meanwhile, the overall effect (involving 455 subjects) showed that there was no significant difference between the two groups (*P*=0.05, MD = −61.80, 95% CI: −124.85 to 1.25).

### 3.7. The Adverse Events of KXC

As illustrated in Table 2, the adverse events were reported in six trials (involving 97 patients), 44 patients in KXC group, and 53 patients in the control group, respectively. The frequent adverse events were gastrointestinal discomfort and abnormal liver function.

## 4. Discussion

Complementary and alternative medicine has been extensively employed in the treatment of inflammatory arthritis [25]. KXC consists of four individual herbs which adhere to the compatibility principle of traditional Chinese medicine: *Tripterygium hypoglaucum*, *Herba Epimedii*, *Fructus Lycii*, and *Semen Cuscuta*. The main component of the KXC is TWHF, which derives from *Tripterygium hypoglaucum* [26, 27]. *Herba Epimedii* was widely used in the clinic to enhance bone fracture healing [28]. *Fructus Lycii* had many pharmacological and biological activities, such as anti-inflammation, antioxidation, antiapoptosis, immune regulation [29]. *Semen Cuscuta* had a protective effect on the reproductive system [30]. In a word, KXC, as an upgrade of TWHF, served as strengthening the therapeutic effect and weakening the side effects of TWHF [31–34].

There were two meta-analyses of KXC in the treatment of RA [35, 36]. Nonetheless, no review protocol was registered. However, one of the meta-analysis lacked data and forest plots [35]. The other meta-analysis did not have strict inclusion criteria [36]. Our analysis included more samples, performed rigorously screening, and had two subgroup analyses.

The results demonstrated that KXC had significant effects in alleviating DMS, ESR, and CRP, whether in two different subgroups or as a whole. These results were consistent with previous meta-analyses [35, 36]. Moreover, TJC was reduced in KXC group, which was similar to the previous meta-analysis [35].

In addition, the study also demonstrated that KXC plus DMARDs group could reduce SJC and RF, but KXC alone or the overall effect of KXC did not lower SJC and RF. However, the previous analyses suggested that KXC could reduce SJC and RF [36]. We considered that the number of RCTs treated with KXC alone is small, which could lead to the difference.

Our study indicated that KXC had no effect on CCP, and previous meta-analyses did not analyze the outcome CCP. The CCP is a diagnostic parameter that cannot reflect disease activity; thereby KXC could not reduce CCP.

The mechanisms of KXC in treating RA have been explored *in vitro* and *in vivo* experiments. Previous research showed that KXC had immunosuppressive, anti-inflammatory, and analgesic effects [37]. Animal experiments demonstrated that KXC downregulated the expression of IL-8 and *γ*IP-10 mRNA, reduced synovial inflammation, alleviated the formation of pannus, decreased joint damage and fibrous tissue proliferation, and promoted tissue repair in the synovium of rats with collagen induced arthritis [38–40]. Additionally, triptolide influenced CD4+ and CD8+ cells distribution in Peyer's patch of DA rats with collagen induced arthritis [41].

However, some limitations of this systematic review should be mentioned. First, all the included trials were conducted in China, which inferred a high risk of selection bias. The results could not be applied to other regions in the world. Second, the majority of the studies published in Chinese were of poor quality. Only one multicenter RCT was confirmed. Third, the limited number of trials (from two to six) and participants included in each subgroup obscured the supportive evidence of KXC for RA. Fourth, the heterogeneity included in each subgroup was also obvious, especially in the outcomes CCP and RF. We consider that the differences in the quality of reports, intervention methods, doses, and duration of treatment lead to heterogeneity. Finally, the most important outcomes (ACR 20, ACR 50) were not reported except for two studies [17, 22]. Considering this, all the results should be carefully interpreted.

## 5. Conclusion

In conclusion, KXC is effective in the treatment of RA through lowering TJC, DMS, ESR, and CRP. KXC may function as anti-inflammation as well as immunosuppression. In a word, KXC, as an upgrade of TWHF, serves as enhancing the efficacy and weakening the side effects of TWHF. KXC, as a novel “phytoimmunosuppressant,” is promising in the treatment of RA and other autoimmune diseases. Considering the low quality of the included trials, more well-designed RCTs are needed before we can recommend KXC to replace or combine with conventional therapies.

## Figures and Tables

**Table 1:** Clinical and demographic characteristics of patients with rheumatoid arthritis.

Study	Number of participants (male/female)		Age (years)		Intervention		Duration	Outcomes
	Experimental	Control	Experimental	Control	Experimental	Control		
Ji [20]	30 (3/27)	30 (5/25)	47.93 ± 11.57	52.30 ± 15.84	KXC 0.6 g bid + control	MTX 10 mg qw, NSAIDs	12 w	①②③④⑤⑥
Jia [22]	30 (9/21)	30 (8/22)	22–70	20–70	KXC 0.6 g tid + control	MTX 10–15 mg qw, folic acid 10 mg qw	12 w	①②③④⑤⑥⑦
Lu et al. [23]	20 (7/13)	20 (8/12)	60–73 (67)	61–72 (66)	KXC 0.3–0.6 g tid + MTX 7.5 mg	MTX 7.5–15 mg qw	24 w	①②③④⑤
Quan and Wang [24]	78	78	54.29 ± 11.08	56.04 ± 11.42	KXC 0.3–0.6 g tid + control	HCQ 0.2 g bid	24 w	④⑤⑥⑦
Zhang and Zhang [21]	36 (13/23)	36 (15/21)	45.6 ± 10.2	46.2 ± 9.8	KXC 0.3 tid + control	MTX 10 mg qw + NSAIDs	12 w	③④⑤⑥
Tian [16]	30 (7/23)	30 (6/24)	61.37 ± 10.43	59.47 ± 11.77	KXC 0.3 g tid + control	LEF 20 mg qd	24 w	④⑤⑥
Liu et al. [18]	50 (4/46)	30 (2/28)	36–62	35–59	KXC 0.6 g tid	LEF 20 mg qn	12 w	④⑤⑥⑦
Lu [19]	30 (11/19)	30 (10/20)	46.31 ± 5.62	47.50 ± 5.71	KXC 0.6 g tid	MTX 10–15 mg qw	6 m	③④⑤
Lin et al. [17]	80 (24/56)	79 (13/66)	52.14 ± 9.70	48.62 ± 13.01	KXC 0.3–0.6 g tid	MTX 10–15 mg qw	12 w	①②③④⑤⑥⑦

*Note*. KXC: Kunxian Capsule; MTX: methotrexate; LEF: leflunomide; HCQ: hydroxychloroquine; ① TJC: tender joint counts; ② SJC: swollen joint counts; ③ DMS: duration of morning stiffness; ④ ESR: erythrocyte sedimentation rate; ⑤ CRP: C-reactive protein; ⑥ RF: rheumatoid factor; ⑦ CCP: anticyclic citrullinated peptide antibody. Values are mean ± standard deviation (SD).

**Table 2:** The adverse events of included studies.

Adverse events	Jia [22]		Lin et al. [17]		Lu et al. [23]		Quan and Wang [24]		Tian [16]		Lu [19]		Total events
	Experiment	Control	Experiment	Control	Experiment	Control	Experiment	Control	Experiment	Control	Experiment	Control	
Number of patients	30	30	80	79	20	20	78	78	30	30	30	30	
Gastrointestinal upset	3	2	7	6	2	2	5	4	4	2	0	2	39
Menstrual disturbance	1	1	1	0	0	0	0	0	0	0	0	0	3
Infection	1	1	0	0	0	2	0	0	2	1	0	2	9
Alopecia	0	0	0	3	0	0	0	0	0	0	0	0	3
Erythra	0	0	0	0	0	0	1	0	0	0	0	0	1
Leukocytopenia	1	2	0	0	1	2	0	0	2	3	0	0	11
ALT/AST elevation	2	2	0	4	0	1	2	2	2	4	0	1	20
Erythrocytopenia	0	0	0	1	0	0	0	0	0	0	0	0	1
Anemia	0	0	0	1	0	0	0	0	2	1	0	0	4
Chest distress	0	0	3	0	0	0	0	0	0	0	0	0	3
Headache and dizziness	0	0	1	0	0	0	1	1	0	0	0	0	3

## Data Availability

The data used to support the findings of this study are available from the corresponding author upon request.
